# Comparison between Conventional and Digital Impressions for Determining Axes and Distances of Three Implants in Straight and Curved Lines: An In Vitro Study

**DOI:** 10.3390/jcm13082352

**Published:** 2024-04-18

**Authors:** Gil Ben-Izhack, Ophir Rosner, Eran Zenziper, Joseph Nissan, Reema Hosary, Diva Lugassy, Asaf Shely

**Affiliations:** 1 Department of Oral Rehabilitation, The Maurice and Gabriela Goldschleger School of Dental Medicine, Faculty of Medical and Health Sciences, Tel Aviv University, Tel Aviv 6997801, Israel; rosnerop@yahoo.com (O.R.); eranzen@gmail.com (E.Z.); nissandr@gmail.com (J.N.); reemahosary@mail.tau.ac.il (R.H.); asafshely@gmail.com (A.S.); 2 Department of Orthodontics, The Maurice and Gabriela Goldschleger School of Dental Medicine, Faculty of Medical and Health Sciences, Tel Aviv University, Tel Aviv 6997801, Israel; diva.lugassy@gmail.com

**Keywords:** CAD-CAM, scan abutment, ISB, implant axis, laboratory scanner, intra-oral scanner, polyether, conventional mpression, Primescan, dental implants, oral rehabilitation

## Abstract

**Background**: In this study, we aimed to compare the effects of conventional and digital impressions on several parameters (inter-implant distance, intra-implant distance, inter-implant axis, and intra-implant axis) of three implants in curved lines and straight lines by using a laboratory scanner (LBS) versus an intra-oral scanner (IOS). **Methods**: Two 3D models were fabricated using a printer, each model with three internal hex implants analogues at the positions of 15#,16#,17# (straight line) and 12#,13#,14# (curved line). Standard intra-oral scan bodies (ISBs) were used, and the two models were scanned using 7 Series dental wings (LBS, reference model), followed by ten scans with Primescan (digital method). Standard Tessellation Language (STL) files were created. Five polyether impressions were taken from each model (straight and curved), and gypsum type 4 models were poured; each model was scanned five times to create a total of 25 STL files for each group (conventional method). The comparison between all the STL files (conventional and digital) was made by superimposition of the STL files on the STL reference model laboratory file using a 3D analyzing software. A Kolmogorov–Smirnov test was performed, followed by Mann–Whitney tests and Wilcoxon signed-rank tests. (*p* < 0.05). **Results**: For the conventional method, the mean errors were significantly higher for the curved line model (12–14) compared to the straight line model (15–17) for most parameters (*p* < 0.05). For the digital method, the mean errors were significantly higher for the curved-line model (12–14) compared to the straight line model (15–17) in half of the parameters (*p* < 0.05). Within the curved line model (12–14) and the straight line model (15–17), the mean errors between the conventional method and the digital method were not significant for most variables. **Conclusions**: The difference between curved lines and straight lines has an impact on the mean error of the conventional method. Both methods are reliable for straight and curved lines in partially dentate situations.

## 1. Introduction

A dental impression produces a negative imprint of oral structures to produce a positive replica of the structures, thus achieving a study model or producing a dental restoration or prosthesis [1]. The accuracy of impressions is a crucial step that affects the success of the treatment, since it should transfer the implant position correctly [2]. Since the accuracy of the impression affects the final restoration, an accurate impression is essential for fabricating a prosthesis with optimal fit [3].

Conventional impression using elastomeric materials is still a widely and commonly used technique for replicating the intra-oral anatomy and transferring information to the dental technician for the fabrication of dental restorations. Another method of transferring the data includes digital impressions, which have several advantages: 3D pre-visualization of the preparation, the patient’s comfort and acceptance, elimination of tray selection procedure, digital storage, minimizing the risk of distortion due to the impression technique, pouring, and disinfecting [4].

A recent systematic review of in vitro studies evaluated the accuracy of dental implant impressions taken with intra-oral scanners (IOS) compared to conventional techniques. It showed that, for implant-supported restorations in cases of partial dentate or single implants, a digital impression is as accurate as a conventional impression. Nevertheless, when examining complete edentulous findings, the results were inconclusive, as several studies found greater accuracy with digital impressions, while others found better accuracy with conventional impressions, and few studies showed no difference between the two techniques [5].

Two types of elastomeric impression materials are commonly available for implant impressions: polyether and polyvinyl siloxane. Currently, polyether (PE) and vinyl polysiloxane (VPS) are considered the materials of choice for taking implant-supported prosthesis impressions [6].

One major limitation of conventional impressions includes inadequate moisture control, which is still an important factor for successful impressions. The results of an investigation into moisture’s effect on the dimensional accuracy and detail reproduction of polyether and polyvinylsiloxane suggest that, in the presence of moisture, polyether impression performs better than PVS and should be used when moisture control is not ideal. It is also reported that polyether impression material can absorb moisture, which causes dimensional expansion [7].

When using the traditional method, an intra-oral impression is taken using an elastomeric impression material, followed by a fabrication of a gypsum model. However, this procedure may cause gagging, pain, and inconvenient taste, and the experience of the dentist may affect the accuracy of the impression. In addition, there are some other disadvantages, such as contamination by saliva and blood and deformation of the impression material or model material [8].

An IOS is a device which enables us to capture and translate three-dimensional (3D) geometric information about intra-oral structures and converts it into a digital data. Daily use of IOS in dental practice has been increasing since IOS first appeared more than 30 years ago; this is due to the ongoing improvements in accuracy, convenience, and the decrease in costs, which makes it more affordable [9].

As CAD/CAM technology appeared, it became possible to scan and create a 3D digital image of an intra-oral structure, including tooth preparation or implant abutment, which can be used to design and fabricate a restoration. Today, in everyday dentistry, this technology is being used for fabricating crowns and bridges (supported both by teeth or implants), but can also be used in the surgery field when planning guided implant surgery, fixing a cleft palate, or performing other jaw surgeries [10].

In prosthodontics, intra-oral scanning has simplified the impression procedure by reducing the number of production steps. This reduces the treatment time, and in some cases, can be as accurate as conventional impressions [11]. Accuracy is affected by the size of the area being scanned, because as the size of the area increases, the accuracy decreases. Therefore, in cases of partial arches, intra-oral scanners are a valid alternative to conventional impressions, but it is still a challenge when trying to capture a complete arch. Many factors influence the accuracy of intra-oral scanning, which can explain the various findings and wide range reported in the literature [12]. This variation might be caused by the different types of IOS technology, operator experience, scanning conditions, scanning technique, and strategy, as well as different types of scan bodies [13].

One main challenge for screw-retained implant prosthesis is achieving a passive fit of the prosthesis superstructure to the implants. Passive fit is an important factor in the long-term success of the prosthesis and the implants. A misfit of the implant-supported superstructure may lead to unfavorable complications, either mechanical or biological in nature. The manifestations of these complications may include fractures of various components in the implant system, pain, marginal bone loss, and even loss of osseointegration. Thus, we want to minimize the misfit and optimize the passive fit, which may increase the survival and success of both the implant and prosthesis [14]. The use of screw retention in combination with stiff materials has even increased the demand for an accurate fit [10].

One can find in the literature several terms which are related to digital files, such as trueness, precision, and accuracy. Precision is defined as the proximity of independent test results to the true value. Trueness is defined as the proximity between independent test results. Accuracy is defined as test results which are close to each other and close to the true value (high precision and high trueness) [15,16].

In everyday dentistry, capturing accurate implant axes is a key factor for achieving passive fit of the restoration, which is clinically significant as it determines the accuracy of the prosthesis, and it also has an influence on the design and manufacturing of the restoration. As technology improves, we can achieve better digital impressions, which allow us to produce more accurate partial dentures. Also, the differences in the passive fit between screw-retained systems and cemented systems has become an insignificant factor [17,18].

In confocal microscopy, a beam of incoming light (the excitation beam) is focused through the microscope’s objective on a small spot inside the tissue, which can be almost as small in diameter as the wavelength of light itself, about 0.5 μm. The same objective gathers the reflected or fluorescent light coming back from the tissue, but unlike conventional light microscopy, this light is projected (like a slide projector) and not directly viewed. In conventional light microscopy, although only a small field of tissue is illuminated at one time, some of the reflected or fluorescent light scatters, which could blur or obscure the image. Confocal microscopy overcomes this problem using a small pinhole aperture in a screen that allows only the light emitted from the desired focal spot to pass through. Any light outside of the focal plane (the scattered light) is blocked by the screen [19].

In the literature, there are many studies and systematic reviews which have compared between digital and conventional impressions for full arches [20,21,22,23,24], and there is repetition in the conclusions, which state that more research needs to be conducted to compare digital and conventional impressions. As we could not find any studies which compared the differences which may occur when scanning a straight line compared to a curved line between the two methods (conventional versus digital), we have decided to investigate this issue in a partial dentate model.

The purpose of this study was to compare conventional and digital impressions for determining the axes and distances of three implants in both straight and curved lines. Our null hypothesis was that no difference would be found between intra-oral scanners and conventional impressions for either group (straight line and curved line)**.**

## 2. Materials and Methods

**Figure 1:**
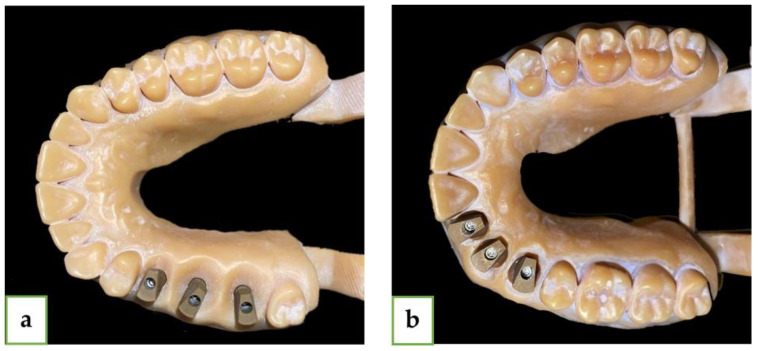
(**a**) Straight line model with MIS ISB. (**b**) Curved line model with MIS ISB.

For this research, we printed two different models using a SolFlex 650 × 350 3D printer (VOCO GmbH, Heidelberg, Germany). Both were made from the same material (resin V-Print model). In both models, we used three standard MIS analogs (M.I.S implants technology, Bar Lev Industrial Park, Misgav, Israel) with diameters of 3.75 mm and lengths of 11.5 mm. In the straight line model, we placed the analogs instead of teeth #15 (1), #16 (2), and #17 (3), and in the curved line model, we placed the analogs instead of teeth #12 (1), #13 (2), and #14 (3). We used a standard MIS (M.I.S implants technology, Bar Lev Industrial Park, Misgav, Israel) intra-oral scan body (ISB), which is made from two pieces of titanium and has an asymmetrical geometry with internal hex connection (Figure 1).

All MIS ISB were screwed to the analogs using an iSD900 screw driver (NSK^®^, Osaka, Japan) for the implant prosthetics at 15 N·cm. The two models were scanned using an extraoral laboratory scanner, 7 Series dental wings (Dental Wings^®^, Montreal QC, Canada). We received a QR file, which was converted to STL file (reference model). This STL file is considered the gold standard in terms of accuracy [15]. Next, we performed ten intra-oral scans (in vitro) of each model with Primescan (CEREC^®^ Primescan; Dentsply Sirona, Milford, DE, USA) using the scanning protocol, which was suggested by the manufacturer and by a single experienced user (A.S.), and we received ten STL files for each model. The comparison of the STL files between each of the 10 intra-oral scans to the extraoral laboratory scan (reference model) was made using digital software PolyWorks Inspector™ 2020 (PolyWorks^®^ Inspector™ 2020; InnovMetric, Québec, QC, Canada) and the best-fit method.

**Figure 2:**
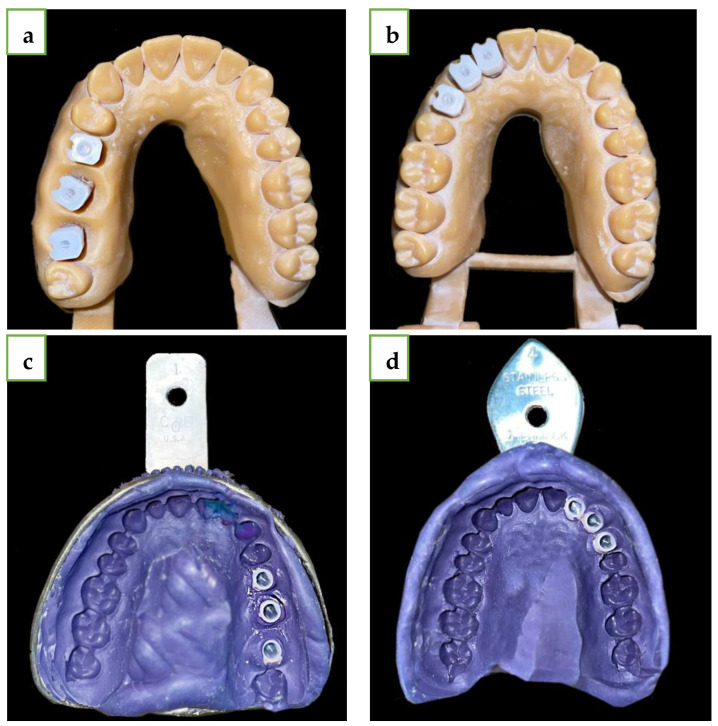
(**a**) Straight line model with MIS snap-ons. (**b**) Curved line model with MIS snap-ons. (**c**) Straight line polyether impression with MIS snap-ons. (**d**) Curved line polyether impression with MIS snap-ons.

After completing the digital scans, the MIS ISB were removed from the model and three standard MIS transfers were screwed to the analogues. One operator (G.B.I.) applied adhesive (Polyether Tray Adhesive, 3M ESPE, Dental Products, St. Paul, MN, USA) to the metal trays, and five monophase polyether (Impregum Penta Soft, 3M ESPE, Dental Products, St. Paul, MN, USA) impressions were taken from each model using a Pentamix device (3M, ESPE) and a closed-tray technique with snap-ons (Figure 2).

**Figure 3:**
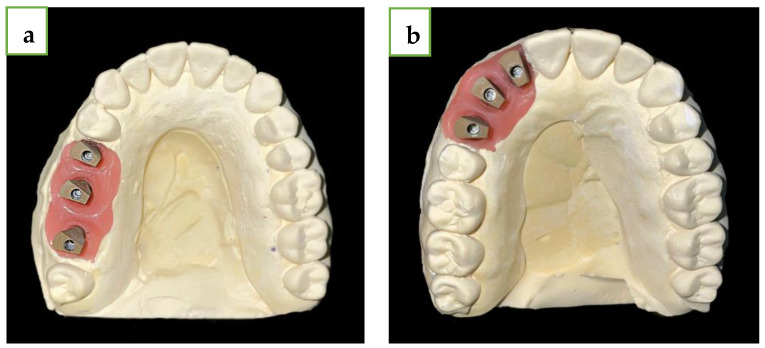
(**a**) Straight line gypsum model with MIS ISB. (**b**) Curved line model with MIS ISB.

For each group, five models were produced from Kromotypo4 type 4 gypsum (LASCOD, Florence, Italy) with MIS standard analogues (M.I.S implants technology, Bar Lev Industrial Park, Misgav, Israel), and the MIS ISB were screwed at 15 N·cm to the models in the same position as before (Figure 3).

**Figure 4:**
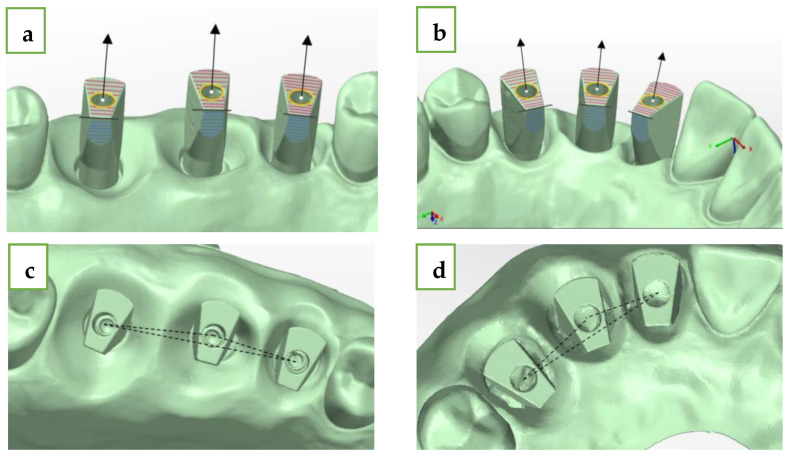
(**a**) Straight-line MIS ISB upper plane, cylinder, axis, central point, side plane, and sideline. (**b**) Curved-line MIS ISB upper plane, cylinder, axis, central point, side plane, and sideline (**c**) Straight-line MIS ISB inter-implant distance. (**d**) Straight-line MIS ISB inter-implant distance.

Next, we performed five intra-oral scans of each of the five gypsum models for each group (a total of twenty-five scans for each group) with Primescan (CEREC^®^ Primescan; Dentsply Sirona, Milford, DE, USA) using the scanning protocol, which was suggested by the manufacturer and by a single experienced user (A.S.), and we received twenty-five STL files for each group (twenty-five for the straight line and twenty-five for the curved line model). The comparison of the STL files between each of the 25 intra-oral scans and the extraoral laboratory scan (reference model) was made using digital software PolyWorks Inspector™ 2020 MIS ISB has a flat surface, which was pointed to the palate (Figure 1). For each model, the mesial abutment was defined as number 1 (#15 and #12), the medial as number 2 (#16 and #13), and the distal as number 3 (#17 and #14). By using PolyWorks|Inspector™ 2020 Software Verification and Measurement, we superimposed (best-fit algorithm) each STL file with the laboratory STL file (reference model) 10 times for the digital method and 25 times for the conventional method for each group (straight and curved) based on the adjacent teeth of the model. To produce the superimposition, we defined key features for the best-fit algorithm. We used several definitions (Figure 4):

- Upper plane (red plane)—top surface of the MIS ISB, which was best-fitted;
- Cylinder (yellow circle)—inner cylinder of MIS ISB, which was best-fitted;
- Axis (black row)—longitudinal axis of MIS ISB, which was best-fitted;
- Central point (white dot)—this point is defined as the intersection between the cylinder and the upper plane of the MIS ISB;
- Palatal side plane (blue plane)—palatal straight plane of the MIS ISB, which was best-fitted;
- Sideline (black line)—this line is defined as the intersection between the upper plane and the palatal side plane for each MIS ISB;
- Inter-implant distance (black intermitted line)—the distance between two central points: distance 1–2 (#15-#16 and #12-#13), distance 2–3 (#16-#17 and #13-#14), and distance 1–3 (#15-#17 and #12-#14). The deviation of each distance from the reference model was calculated by subtraction between the two results.

**Figure 5:**
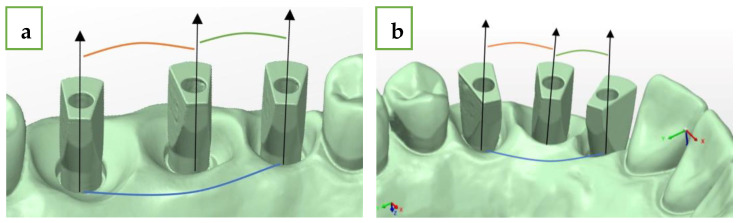
(**a**) Straight-line MIS ISB delta axes. (**b**) Curved-line MIS ISB delta axes.

The angle formed between two longitudinal axes of each MIS ISB is defined as: delta axis 1–2 (#15-#16 and #12-#13), delta axis 2–3 (#16-#17 and #13-#14), and delta axis 1–3 (#15-#17 and #12-#14) (Figure 5):

h. Delta axis 1–2 (green)—defined as the angle formed between the axis of the mesial (#15 and #12) and middle (#16 and #13) MIS ISB;
i. Delta axis 2–3 (orange)—defined as the angle formed between the axis of the middle (#16 and #13) and distal (#17 and #14) MIS ISB;
j. Delta axis 1–3 (blue)—defined as the angle formed between the axis of the mesial (#15 and #12) and distal (#17 and #14) MIS ISB.

The deviation of each angle from the reference model was calculated by subtraction between the two results.

**Figure 6:**
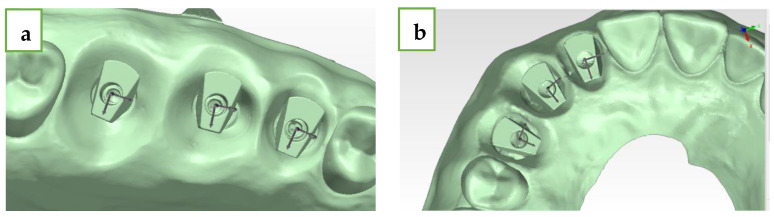
(**a**) Straight-line MIS ISB x,y,z axes. (**b**) Curved-line MIS ISB x,y,z axes.

For both the straight and curved MIS ISB of the reference model, we defined three dimensional axes (x,y,z). The origin of this system is located in the center of the upper plane (Figure 6):

*X*-axis (red)—buccal–palatal plane; buccal is the positive direction;
*Y*-axis (green)—mesial–distal plane; distal is the positive direction;
*Z*-axis (blue)—occlusal–gingival plane; occlusal is the positive direction.

The superimposition was performed using PolyWorks|Inspector™ 2020 Software Verification and Measurement. We superimposed (best-fit algorithm) each reference model’s STL file (laboratory scan) with each intra-oral scan’s STL file for both the conventional method and the digital method based on the adjacent teeth of the model. Due to the superimposition process, we were able to measure all the axes. Subsequently, we were able to extract the key features from each MIS ISB and calculate the spatial characterization of the ISB relative to the reference as follows.

For all the measurements, we defined the central point as a key feature. The shift of the MIS ISB between the intra-oral scans (conventional or digital) and the laboratory scan (reference model) was based on the location of the central point (white dot). The following calculation was made:

$$D\;\mathrm{central}\;{\mathrm{point}}_{1,2,3}=\sqrt{X^{2}+Y^{2}+Z^{2}}$$

Delta axis _1,2,3_—calculated as the three-dimensional angle between each longitudinal axis of the laboratory scan (reference model) and its counterpart longitudinal axis of the intra-oral scan (conventional and digital).

Intra-implant distance (mm)—the three-dimensional changes between the D central points of each MIS ISB intra-oral scan (conventional and digital) and the D central point of the laboratory scan (reference point).

Inter-implant distance (mm)—the three-dimensional changes between the D central points of two MIS ISB intra-oral scans (conventional and digital) and the results of the same measurements of the laboratory scan (reference point).

Intra-implant angle (angle)—the three-dimensional changes between the delta-axis in each single MIS ISB intra-oral scan (conventional or digital) and the delta-axis of the laboratory scan (reference point).

Inter-implant angle (angle)—the three-dimensional changes between the delta-axis of two MIS ISB intra-oral scans (conventional and digital) compared to the results of the same measurements of the laboratory scan (reference point).

A statistical analysis was performed using the Statistical Package for Social Sciences for Windows Release 23.0 (SPSS Inc., Chicago, IL, USA). A Kolmogorov–Smirnov test indicated to us whether the distribution was normal or not. If normal, we used independent-sample T tests to compare between the groups (straight versus curved) and paired-sample T tests to compare within the groups (conventional versus digital). If the distribution is not normal, we will use Mann–Whitney tests for independent variables to compare between the groups (straight versus curved) and Wilcoxon signed-ranks tests to compare within the groups (conventional versus digital). The statistical significance level for this work was *p* < 0.05.

## 3. Results

We used a Kolmogorov–Smirnov test on the study variables, and the results indicated no normal distribution (*p* < 0.05). A sensitivity power analysis was performed using G*power to calculate whether the size of the effect our study was sensitive enough to detect. A Mann–Whitney test with two groups, *n* = 25 and *n* = 10, would be sensitive to the effect of Cohen’s d = 0.97 with 80% power (α = 0.05, two tailed). Mann–Whitney tests were used for comparison between the groups, i.e., the straight model (15–17) and the curved model (12–14). Wilcoxon signed-rank tests were used for comparison within the groups to evaluate the differences between the conventional method (polyether) and the digital method (intra-oral scanning).

### 3.1. Between Groups

**Figure 7:**
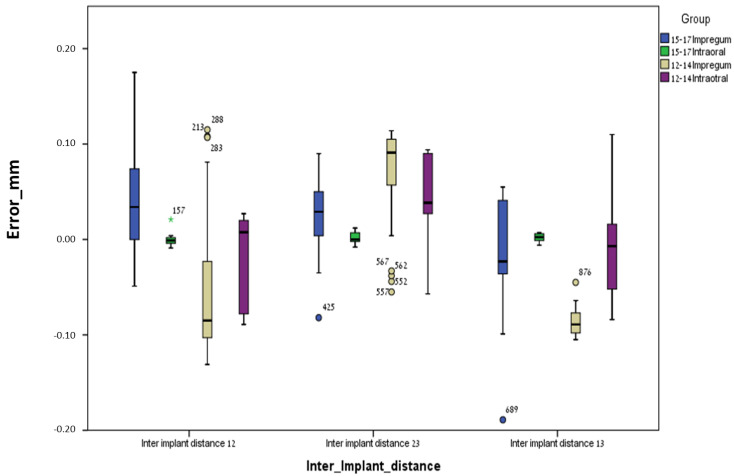
Inter-implant distance error and median for all groups.

**Figure 8:**
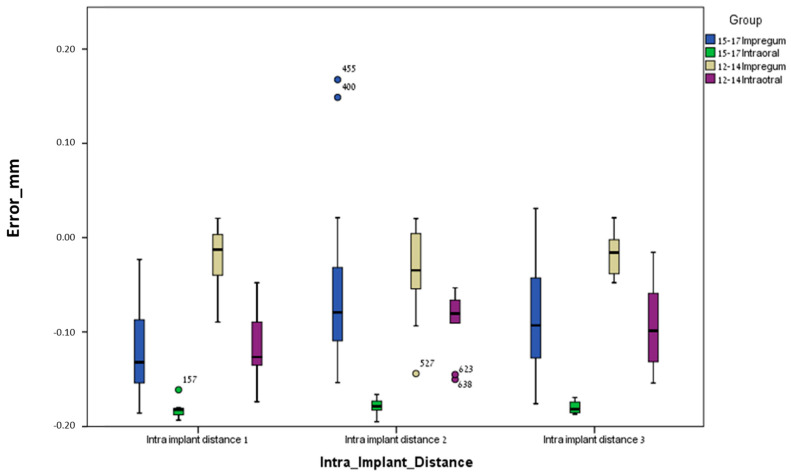
Intra-implant distance (central point 1,2,3) error and median for all groups.

**Figure 9:**
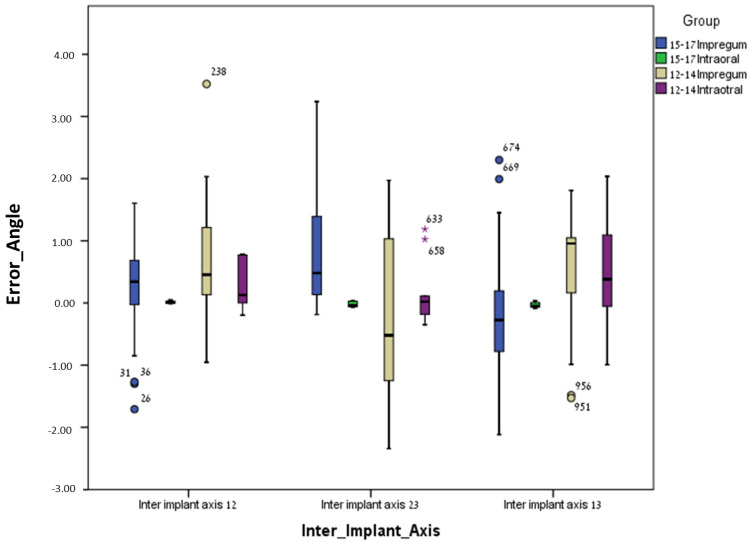
Inter-implant axis (delta-axes 12,23,13) error and median for all groups. Outliers identified by asterisks (*).

**Figure 10:**
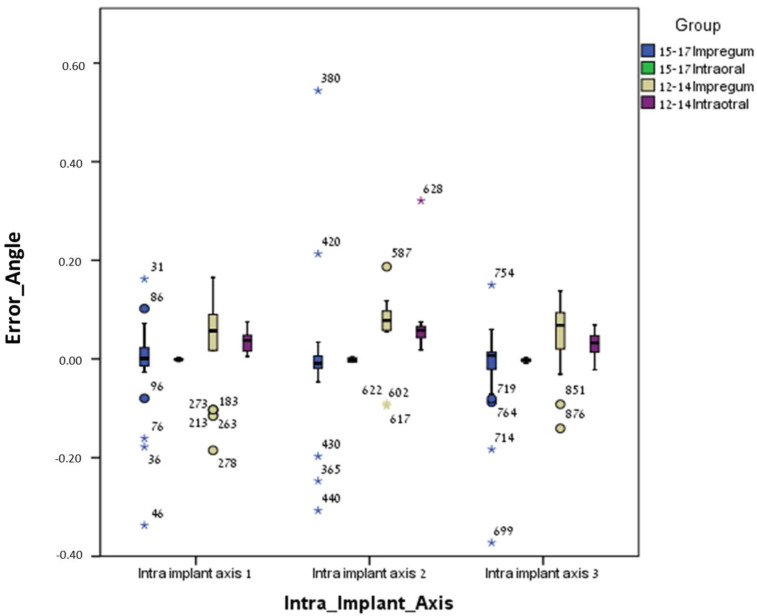
Intra-implant axis (delta axis 1,2,3) error and median for all groups. Outliers identified by asterisks (*).

For the conventional method (polyether), the mean errors were significantly higher (less accurate) for the curved line model (12–14) compared to the straight line model (15–17) regarding most parameters: inter-implant distance 12 (*p* = 0.0005), inter-implant distance 23 (*p* = 0.001), inter-implant distance 13 (*p* = 0.0005), intra-implant distance 1 (*p* = 0.0005), intra-implant distance 2 (*p* = 0.014), intra-implant distance 3 (*p* = 0.0005), intra-implant axis 1 (*p* = 0.035), intra-implant axis 2 (*p* = 0.0005), intra-implant axis 3 (*p* = 0.001), inter-implant axis 23 (*p* = 0.007), and inter-implant axis 13 (*p* = 0.006). Only one parameter, inter-implant axis 12, was not significant (*p* = 0.128) (Table 1 and Table 2 and Figure 7, Figure 8, Figure 9 and Figure 10).

For the digital method (intra-oral scanner), the mean errors were significantly higher (less accurate) for the curved line model (12–14) compared to the straight line model (15–17) for the following parameters: inter-implant distance 23 (*p* = 0.023), intra-implant distance 1 (*p* = 0.0005), intra-implant distance 2 (*p* = 0.005), intra-implant distance 3 (*p* = 0.0005), intra-implant axis 1 (*p* = 0.0005), intra-implant axis 2 (*p* = 0.0005), and intra-implant axis 3 (*p* = 0.023). Five parameters out of twelve (almost half), i.e., inter-implant axis 12 (*p* = 0.089), inter-implant axis 23 (*p* = 0.481), inter-implant axis 13 (*p* = 0.063), inter-implant distance 12 (*p* = 0.739) and inter-implant distance 13 (*p* = 0.529), were not significant (Table 1 and Table 2 and Figure 7, Figure 8, Figure 9 and Figure 10).

### 3.2. Within Groups

Within the curved line model (12–14), the mean errors between the conventional method (polyether) and the digital method (intra-oral scanner) were not significant regarding most variables (*p* > 0.128), except for inter-implant distance 13 (*p* = 0.007) and intra-implant distance 1 (*p* = 0.008), in which the mean errors of the conventional method (polyether) were significantly higher (less accurate) than those of the digital method (intra-oral scanner).

Within the straight line model (15–17), the mean errors between the conventional method (polyether) and the digital method (intra-oral scanner) were not significant regarding most variables (*p* > 0.074), except for intra-implant distance 1 (*p* = 0.007), intra-implant distance 2 (*p* = 0.005), intra-implant distance 3 (*p* = 0.005), and inter-implant axis 23 (*p* = 0.017), in which the mean errors of the conventional method (polyether) were significantly higher (less accurate) than those of the digital method (intra-oral scanner).

## 4. Discussion

In this in vitro study, we evaluated the differences (mean error) in several parameters (inter-implant distance, intra-implant distance, inter-implant angle and intra-implant angle) between the conventional elastomeric impression (polyether) method and the digital impression method with IOS (primescan). We utilized two different models with three implants: a straight line model (#15,#16,#17) and a curved line model (12#,13#,14#). The evaluation of the STL files was performed by superimposition to a reference STL file, which was created using a laboratory scanner (reference model).

Our null hypothesis was partially rejected, as there were significant differences between the groups (the straight line model compared with the curved line model). For the conventional method (polyether), the mean errors were significantly higher for the curved line model compared to straight line model for most parameters, except one, which was not significant. For the digital method (Primescan), the mean errors were significantly higher for the curved line model compared to the straight line model in approximately half of the parameters, as the other parameters were not significant.

These results indicate that, with the conventional polyether impression, the curved line group exhibited mean errors which were much higher compared to the straight line group, while in the digital impression, the mean errors were higher in only half of the parameters. We know from the literature that the conventional impression method accuracy relies on every phase of the impression-taking process, including the impression material and stone casts, which must be carried out precisely to achieve the best fit. Contrastingly, dental workflow with CAD/CAM systems requires fewer steps, as the number of error sources is fewer than in the conventional method [25,26].

Although there were more parameters with no significant differences in the digital group, they were not significant. Many studies in the literature have shown that there are no differences in partial dentate between digital and conventional impressions [4,27,28], while some have shown that conventional methods are superior [29,30,31].

In the conventional group, we used a closed tray with a Snap-On system. Lee et al., in their systematic review, showed that no differences were found between the pick-up and transfer techniques in most of the studies when dealing with cases of three or fewer implants for both polyether and vinyl poly siloxane materials. For four implants or more, most of the studies showed that the pick-up technique has higher accuracy [3] In our study, because both groups included three implants and we used polyether, it was acceptable to compare the closed-tray technique with Snap-Ons to the digital method. We do know that one of the disadvantages of the Snap-On technique is that the three-dimensional position of the components might change during tray removal, especially when multiple nonparallel implants are present [32].

In this study, we used MIS ISB, which has special trapezoid geometry and a relatively large surface area, and can reduce inter-implant distance when scanning. In our previous study, we concluded that the geometry of the scan abutments has an impact on the inter-implant distance in MIS ISB [33].

Regarding the inter-implant distance, it can be seen from the results that the highest mean error occurred with polyether technique for all groups (13,12,22). We know from previous publications that there is a connection between inter-implant distance and scanning errors; as the distance increases, the distortion also increases. These reports were regarding complete edentulous arches [13,34,35]. In this research, our results indicate that the curve has an impact on the error, as we measured only three implants and not a complete edentulous arch. Because most of the studies today focus on full arches, we wanted to take a step back and examine whether, if we check only three implants on straight and curved lines between teeth, it has an impact—this was the rationale of this study. We also know that, when there are no teeth as landmarks in complete edentulous arches, the distortions increase [36,37]. In our case, there were many teeth for landmarks, and we still perceived differences between the straight and curved line groups.

Regarding the inter-implant angle, the results indicate that the polyether had the highest mean error for all groups (13,12,22). A recent study by Osta et al. used a model with implants #14 and #24, #15 and #25, and #17 and #27 (free saddles). Impressions were taken with polyether and five different IOS, among them Primescan, and the digital methods achieved better accuracy. They showed that, for partial arch segments, the IOS is a valid alternative to the conventional method [38]. In our study, when comparing the conventional method to the digital method, most variables did not significantly differ, but for some, the mean errors with the conventional method were higher. There are already studies which have demonstrated that, for short spans with no more than 4–5 implants, IOS can be used and achieves acceptable results [12,27,39].

A study by Andriessen et al. showed that, when using a 14.8 mm implant, the intra-implant angle must be limited to 0.194 degrees, because it creates a 50 μm difference at the apex of the implant [40]. Hence, we want to achieve an intra-implant angle of less than 0.2 degrees of and an inter-implant angle of less than 0.4 degrees for a physiologic bone strain. In this study, we did not perceive any deviation in the intra-implant distance, which exceeded 0.194 degrees for all implants with all methods. For the inter-implant distance, we exceeded 0.4 degrees in curved line #13 for both polyether and Primescan, in polyether straight line #23, and in polyether curved line #12.

In this study, we used a method to measure the inter-implant angle, intra-implant angle, inter-implant distance, and intra-implant distance of three implants in straight and curved lines using both a digital method and the conventional method, and compared them with a laboratory scanner. There is still a lack of information in the literature regarding the differences between the digital and conventional methods when taking impressions of a short span, either in a straight line or a curved line.

Finally, there limitations to this in vitro study, as this model does not assume clinical validity of impressions in the oral environment where there is saliva or blood and the access in the clinic is less convenient [41]. However, due to the in vitro model, we were able to compare a straight line model to a curved line one.

## 5. Conclusions

In the implantology field, it has long been known that the conventional method is valid and clinically accepted. Our findings reveal that within groups (straight and curved lines), in most of the variables, there were no differences between the conventional and digital methods, and when there were differences in mean errors, they were higher for the conventional method. Between groups, for the conventional method, the mean error was significantly higher for the curved line group, and for the digital method, only half of the parameters demonstrated significantly higher mean errors. The difference between a curved line and a straight line has an impact on the mean error of the conventional method. When comparing a straight line to a curved line, the digital method is preferred. It can be concluded that both methods are reliable for straight and curved lines in partially dentate situations.

## Figures and Tables

**Table 1:** Median, range, P25 (25 percentile), P50 (50 percentile), and P75 (75 percentile) of inter-implant distance 12,23,13; intra-implant distance 1,2,3; intra-implant axis 1,2,3; and inter-implant axis 12,23,13 of the conventional method (polyether, *n* = 25) and the digital method (intra-oral scanner, *n* = 10) for a 15–17 model.

Conventional Method 15–17 (Polyether)					Digital Method 15–17 (Intra-Oral Scanner)			
	Median	P25	P50	P75	Median	P25	P50	P75
Inter-implant distance 12 (mm)	0.081	0.072	0.081	0.086	−0.039	−0.051	−0.039	−0.022
Inter-implant distance 23 (mm)	−0.133	−0.138	−0.133	−0.124	0.013	0.000	0.013	0.021
Inter-implant distance 13 (mm)	−0.046	−0.052	−0.046	−0.038	−0.020	−0.034	−0.020	−0.006
Intra-implant distance (Central point 1) (mm)	0.057	0.049	0.057	0.064	0.019	0.012	0.019	0.025
Intra-implant distance (Central point 2) (mm)	0.135	0.132	0.135	0.140	0.047	0.040	0.047	0.059
Intra-implant distance (Central point 3) (mm)	0.021	0.016	0.021	0.027	0.073	0.065	0.073	0.083
Intra-implant axis Delta axis 1 (angle)	0.311	0.244	0.311	0.344	0.355	0.245	0.355	0.495
Intra-implant axis Delta axis 2 (angle)	1.463	1.410	1.463	1.498	1.675	1.442	1.675	2.024
Intra-implant axis Delta axis 3 (angle)	0.142	0.094	0.142	0.189	1.975	1.832	1.975	2.256
Inter-implant axis Delta axis 12 (angle)	0.407	0.300	0.407	0.469	−0.511	−0.810	−0.511	−0.065
Inter-implant axis Delta axis 23 (angle)	−0.995	−1.067	−0.995	−0.947	0.250	−0.111	0.250	0.540
Inter-implant axis Delta axis 13 (angle)	−0.217	−0.266	−0.217	−0.157	−1.724	−2.070	−1.724	−1.459

**Table 2:** Median, range, P25 (25 percentile), P50 (50 percentile), and P75 (75 percentile) of inter-implant distance 12,23,13; intra-implant distance 1,2,3; intra-implant axis 1,2,3; and inter-implant axis 12,23,13 of the conventional method (polyether, *n* = 25) and the digital method (intra-oral scanner, *n* = 10) for a 12–14 model.

Conventional Method 12–14 (Polyether)					Digital Method 12–14 (Intra-Oral Scanner)			
	Median	P25	P50	P75	Median	P25	P50	P75
Inter-implant distance 12 (mm)	0.081	0.072	0.081	0.086	−0.039	−0.051	−0.039	−0.022
Inter-implant distance 23 (mm)	−0.133	−0.138	−0.133	−0.124	0.013	0.000	0.013	0.021
Inter-implant distance 13 (mm)	−0.046	−0.052	−0.046	−0.038	−0.020	−0.034	−0.020	−0.006
Intra-implant distance (Central point 1) (mm)	0.057	0.049	0.057	0.064	0.019	0.012	0.019	0.025
Intra-implant distance (Central point 2) (mm)	0.135	0.132	0.135	0.140	0.047	0.040	0.047	0.059
Intra-implant distance (Central point 3) (mm)	0.021	0.016	0.021	0.027	0.073	0.065	0.073	0.083
Intra-implant axis Delta axis 1 (angle)	0.311	0.244	0.311	0.344	0.355	0.245	0.355	0.495
Intra-implant axis Delta axis 2 (angle)	1.463	1.410	1.463	1.498	1.675	1.442	1.675	2.024
Intra-implant axis Delta axis 3 (angle)	0.142	0.094	0.142	0.189	1.975	1.832	1.975	2.256
Inter-implant axis Delta axis 12 (angle)	0.407	0.300	0.407	0.469	−0.511	−0.810	−0.511	−0.065
Inter-implant axis Delta axis 23 (angle)	−0.995	−1.067	−0.995	−0.947	0.250	−0.111	0.250	0.540
Inter-implant axis Delta axis 13 (angle)	−0.217	−0.266	−0.217	−0.157	−1.724	−2.070	−1.724	−1.459

## Data Availability

The data presented in this study are available upon request from the corresponding author.
